# Integrated single-cell and bulk-tissue transcriptomic analysis reveals an epithelial-stromal inflammatory microenvironment in biliary atresia

**DOI:** 10.3389/fped.2026.1948945

**Published:** 2026-09-21

**Authors:** Songxian Lu, Zhansheng Wang, Xin Li

**Affiliations:** Department of Pediatric Surgery, The Third Affiliated Hospital of Zhengzhou University, Zhengzhou, China

**Keywords:** biliary atresia, cell-cell communication, cholangiocyte, fibroblast, liver microenvironment, single-cell transcriptomics

## Abstract

**Background:**

Biliary atresia is a severe fibroinflammatory cholangiopathy of infancy. Its pathological process involves biliary epithelial injury, immune activation, and intrahepatic stromal remodeling, but the cellular interactions linking these processes remain incompletely defined. Using public single-cell and bulk-tissue transcriptomic cohorts, this study systematically characterized disease-associated alterations across liver cell populations in biliary atresia and further evaluated their consistency in independent cohorts.

**Methods:**

Public single-cell liver data (GSE176189) and three independent bulk-tissue cohorts (GSE122340, GSE300457, and GSE46960) were analyzed. The single-cell cohort contained 119,538 cells after filtering, including 89,118 cells from biliary atresia samples and 30,420 cells from choledochal cyst samples. Disease-associated genes were identified within major cell populations, used to construct cell-origin microenvironment signatures, and evaluated across bulk cohorts.

**Results:**

Broad cell-type annotation identified cholangiocytes, macrophages, T cells, endothelial cells, fibroblasts, and other liver-resident or immune populations. Based on biliary atresia-upregulated genes from five key cell populations, a 219-gene pathological microenvironment signature was constructed. Cell-type-specific enrichment analysis highlighted immune, antiviral, migration, and matrix-related biological processes, although the fibroblast component was based on fewer genes and was interpreted cautiously. In three bulk-tissue cohorts, the composite microenvironment score was elevated in biliary atresia samples, with areas under the receiver operating characteristic curve of 0.993, 0.782, and 0.798, respectively. Cell-communication analysis suggested candidate MIF, GALECTIN, CLEC, PTN, NOTCH, and CXCL signaling axes, which require spatial or experimental validation.

**Conclusion:**

These findings support a multi-cellular inflammatory microenvironment in biliary atresia liver and nominate epithelial-immune-stromal communication axes for future validation, while recognizing that the single-cell comparison used choledochal cyst rather than normal liver as the control condition.

## 1 Introduction

Biliary atresia is one of the most important cholestatic diseases of infancy and a leading indication for pediatric liver transplantation. Clinically, it usually has an early onset and rapid progression, and cholestasis may progress to biliary cirrhosis and end-stage liver disease within a short period (1). Clinical outcomes in biliary atresia are influenced by multiple factors, including the timing of diagnosis, the timing of Kasai portoenterostomy, the degree of intrahepatic inflammation, and the progression of fibrosis. Therefore, ductal obstruction alone is insufficient to explain the disease course (2).

Previous studies have suggested that biliary atresia has a multifactorial etiology, potentially involving viral infection, immune dysregulation, abnormal bile duct development, genetic susceptibility, and impaired repair after biliary epithelial injury (3). During disease progression, the biliary epithelium is not merely a passive target of injury; it may contribute to local inflammatory amplification by secreting chemokines, expressing antigen-presentation-related molecules, and activating fibrosis-related pathways (4).

Intrahepatic inflammation and fibrotic remodeling are important histological bases of poor prognosis in biliary atresia. Accumulating evidence indicates close links among portal immune-cell infiltration, ductular reaction, and stromal deposition, and these links may influence postoperative bile drainage and native liver survival (5, 6). Therefore, understanding biliary atresia from the perspective of multicellular interactions may more closely reflect the nature of the disease than focusing on a single cell type or pathway.

Single-cell studies have provided a new framework for interpreting the pathobiology of biliary atresia. Prior work has shown that abnormal cholangiocyte states exist in biliary atresia liver tissue and that these states, together with immune and stromal cells, form a local pathological niche (7). In parallel, bulk-tissue transcriptomic studies have identified molecular signatures that can predict clinical outcomes, suggesting that immune and fibrotic states within the liver are detectable across samples (8, 9).

Although previous studies have laid an important foundation, three questions remain to be clarified. First, which broad cell populations carry the major disease-associated transcriptional alterations in biliary atresia? Second, can cell-derived signatures identified at the single-cell level be supported in independent bulk-tissue cohorts? Third, are there communication axes among epithelial, immune, endothelial, and stromal cells that should be prioritized for validation? To address these questions, we performed an integrative analysis to define reproducible microenvironment signatures and nominate candidate axes suitable for subsequent histological and functional validation.

## 2 Materials and methods

### 2.1 Data sources and study design

Public human liver transcriptomic cohorts were included in this study. The primary single-cell cohort was GSE176189, in which biliary atresia liver samples were compared with choledochal cyst liver samples. The choledochal cyst samples were treated as non-BA biliary disease controls rather than normal liver controls, and findings from this comparison were therefore interpreted as BA-associated relative to choledochal cyst rather than strictly BA-specific. Bulk-tissue cohorts included GSE122340, GSE300457, and GSE46960. GSE122340 included 171 biliary atresia samples and 7 normal liver samples; GSE300457 included 25 biliary atresia samples and 20 Ctrl liver samples; and GSE46960 included 64 biliary atresia samples, 7 normal liver controls, and 14 diseased liver controls, while 10 internal normalization samples were excluded from the disease comparison (10, 11). The overall study design is shown in Figure 1.

**Figure 1:**
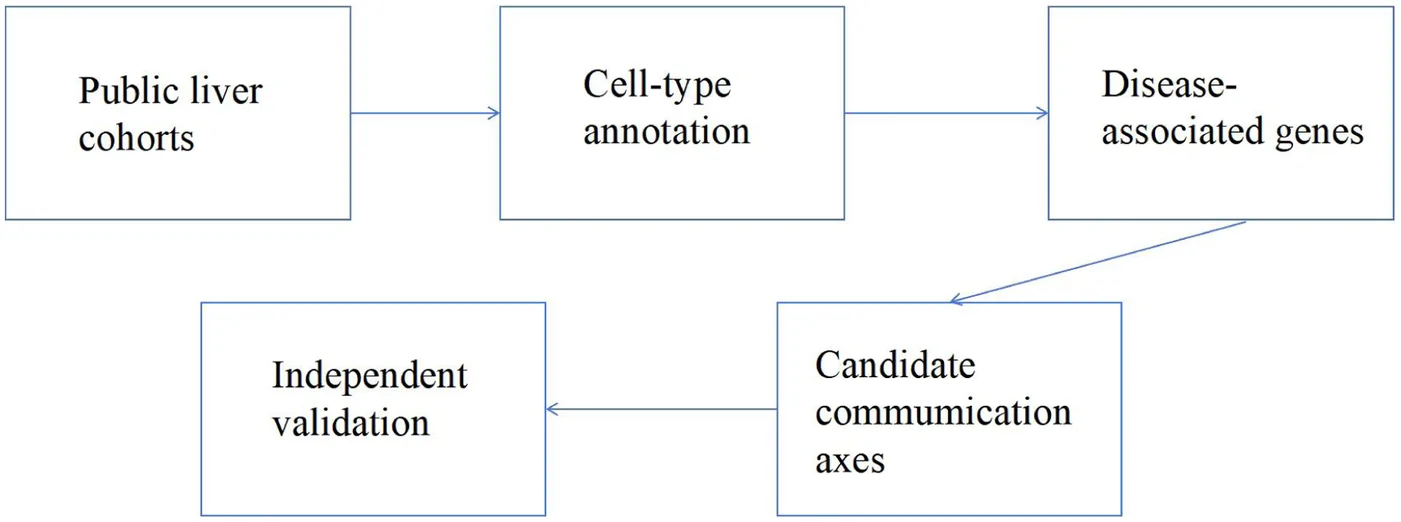
Schematic overview of the study design. A public biliary atresia single-cell cohort was used to identify liver cell populations and disease-associated gene alterations; independent bulk-tissue cohorts were used to evaluate the consistency of cell-origin signatures; and candidate mechanisms were then inferred from biological function, cell-state, and cell-communication analyses.

### 2.2 Single-cell filtering, dimensionality reduction, clustering, and cell-type annotation

Raw single-cell expression matrices were used to create sample-level objects, and genes detected in at least three cells were retained. At the cell level, cells with nFeature_RNA between 200 and 7,500 and a mitochondrial gene percentage of no more than 20% were retained. Expression normalization, variable-feature identification, data scaling, and principal component analysis were then performed. The first 30 principal components were used for UMAP visualization, nearest-neighbor graph construction, and clustering, with the clustering resolution set to 0.6. Broad cell types were assigned at the cluster level according to the average expression of canonical liver and immune cell marker genes, with additional correction based on core cholangiocyte markers. Cholangiocyte markers included KRT7, KRT8, KRT18, KRT19, EPCAM, SOX9, and MUC1; fibroblast markers included COL1A1, COL1A2, DCN, and LUM; macrophage markers included LYZ, CD68, C1QA, and C1QB. T cells, B cells, NK cells, endothelial cells, hepatocytes, and smooth muscle cells were annotated with reference to CD3D/CD3E/TRAC, MS4A1/CD79A, NKG7/GNLY, PECAM1/VWF/KDR, ALB/APOA1/TTR, and ACTA2/TAGLN/MYH11, respectively (12–15). Canonical marker expression was summarized together with the UMAP annotation to support the broad cell-type assignments.

### 2.3 Identification of disease-associated genes and construction of signature gene sets

Disease-associated gene changes between biliary atresia and choledochal cyst controls were compared separately in cholangiocytes, macrophages, T cells, endothelial cells, and fibroblasts. Comparisons were performed within each cell population using a Wilcoxon rank-sum testing framework. Candidate genes were required to be detected in at least 10% of cells, and genes with an average log2 fold-change threshold greater than 0.1 were retained in the initial comparison. To reduce the influence of extreme differences in cell numbers, no more than 2,000 cells were included from each comparison group. More stringent criteria were used for reporting results and constructing signatures, namely an adjusted *P* value < 0.05 and an absolute average log2 fold change > 0.25. For each cell population, BA-upregulated genes were ranked by adjusted *P* value and effect size, and up to 50 genes were selected to give each cell-origin signature a comparable maximum contribution while preserving interpretability. When fewer than 50 genes passed the reporting threshold, all eligible genes were retained. Therefore, the fibroblast signature contained 19 genes, reflecting the strict filtering threshold and the small number of control fibroblasts rather than evidence that fibroblasts were biologically inactive.

### 2.4 Signature scoring and statistical analysis in bulk-tissue cohorts

To assess cross-cohort consistency, the above gene sets were projected onto three bulk-tissue cohorts. For each cohort, the gene expression matrix was transformed into gene-level Z scores. For each sample, the cell-origin signature score was calculated as the mean Z score of detectable genes within the corresponding gene set, and a score was computed when at least three genes were detected. The composite microenvironment score was defined as the mean of five cell-origin signature scores: cholangiocyte, endothelial cell, fibroblast, macrophage, and T cell. Between-group comparisons were performed using the Wilcoxon rank-sum test, followed by Benjamini-Hochberg correction within each cohort. Discriminative performance was represented by the area under the receiver operating characteristic curve. A rank-based single-sample enrichment sensitivity analysis was additionally used to examine whether the composite-score separation was dependent on the mean Z-score framework.

### 2.5 Cell-cell communication analysis

Cell-cell communication analysis was performed separately within the biliary atresia and choledochal cyst groups based on ligand-receptor expression relationships. To reduce domination by very abundant cell populations and to make source-target comparisons more comparable across cell types, up to 500 cells were randomly sampled from each disease group-cell type combination, with the random seed set to 20260615. This sampling strategy reduces imbalance but may also decrease sensitivity for rare cell populations. The analysis was restricted to three interaction categories: Secreted Signaling, ECM-Receptor, and Cell-Cell Contact. Communication probabilities were calculated using the triMean approach, and cell-group interactions involving fewer than 10 cells were filtered. Differences between the two groups were then compared in terms of significant ligand-receptor interactions, source-target cell directions, and pathway-level signals (16). These results were interpreted as co-expression-based communication hypotheses rather than evidence of spatial proximity, pathway activation, or causal signaling.

### 2.6 Cell-state trajectory analysis

Trajectory analysis focused on cholangiocytes and fibroblasts to examine whether continuous state differences existed within these two cell populations. The analysis was implemented with Monocle3. Each cell population was converted into a cell-data-set object, and up to 30 dimensions were used for preprocessing, dimensionality reduction, cell clustering, and principal graph learning. No root node or experimentally confirmed starting state was specified. Therefore, the trajectory plots were used only to describe transcriptional-state heterogeneity and were not interpreted as a definite temporal order, developmental lineage, or disease-progression direction (17–19).

### 2.7 Biological function enrichment analysis

Biological function interpretation was performed at two levels. First, BA-upregulated genes from each key cell population were analyzed separately using Gene Ontology biological process enrichment analysis after conversion of gene symbols to Entrez IDs. Second, the combined 219-gene pathological microenvironment signature was assessed to summarize shared biological themes across cell origins. Enriched terms were interpreted primarily according to adjusted *P*-value ranking and gene count, with emphasis on immune activation, antiviral responses, leukocyte differentiation, epithelial stress, stromal-cell communication, and fibrosis-related processes (20).

## 3 Results

### 3.1 Biliary atresia liver tissue contains complex epithelial, immune, endothelial, and stromal cell populations

The primary single-cell cohort included in this study contained 119,538 liver cells, including 89,118 cells from biliary atresia samples and 30,420 cells from choledochal cyst samples. Broad cell-type annotation identified B cells, cholangiocytes, endothelial cells, fibroblasts, hepatocytes, macrophages, NK cells, smooth muscle cells, and T cells. Canonical marker expression supported these broad annotations, including KRT19/EPCAM in cholangiocytes, COL1A1/DCN in fibroblasts, LYZ/C1QA in macrophages, PECAM1/VWF in endothelial cells, CD3D/TRAC in T cells, and MS4A1/CD79A in B cells (Figure 2).

**Figure 2:**
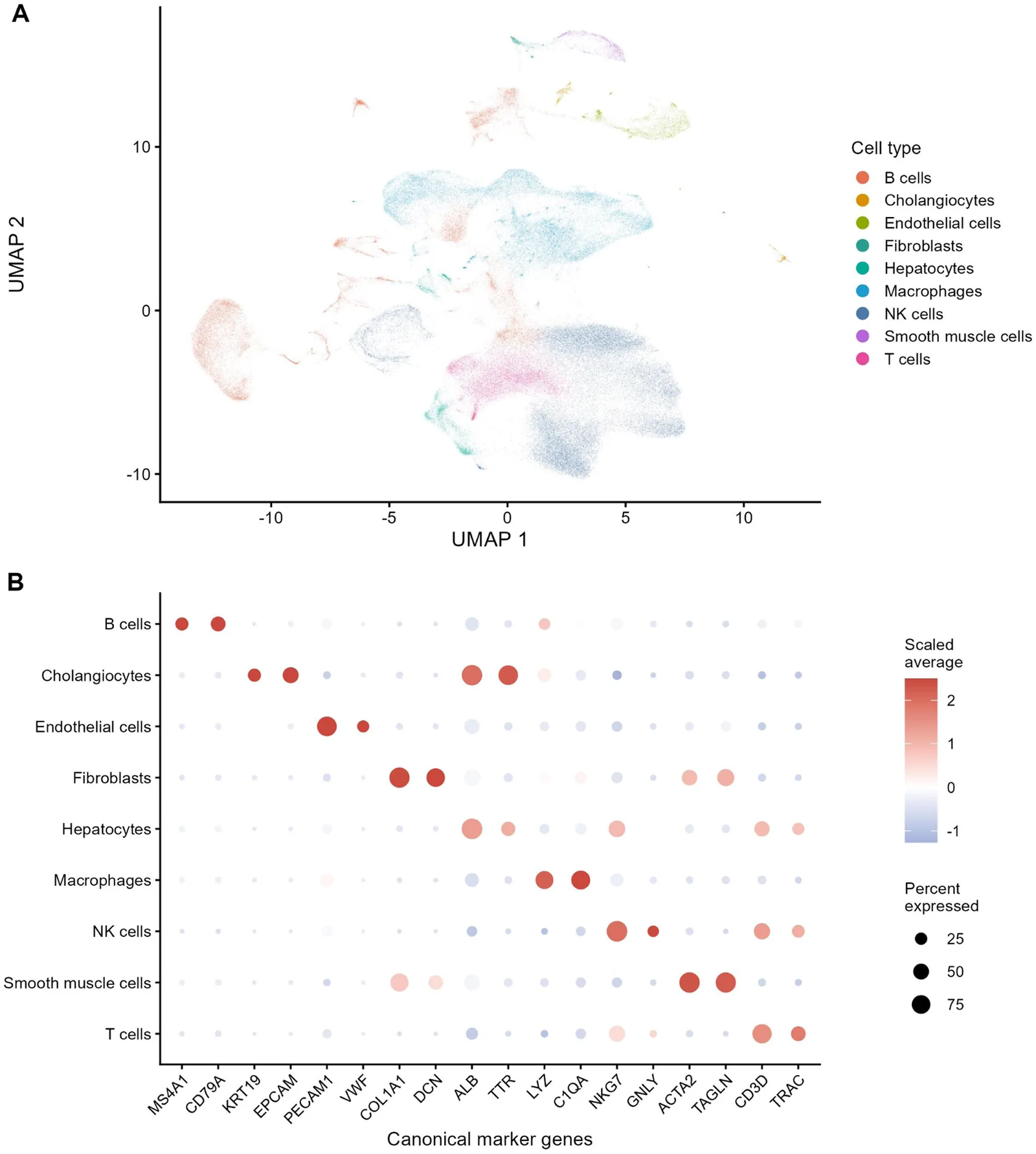
Broad cell-type annotation and canonical marker expression in the single-cell cohort. **(A)** UMAP distribution of major liver and immune cell populations in biliary atresia and choledochal cyst samples, with colors corresponding to the cell types shown in the legend. **(B)** Dot plot of representative canonical marker genes supporting the broad cell-type annotation.

In biliary atresia samples, NK cells, macrophages, B cells, and T cells were relatively abundant, with 31,485, 19,049, 17,716, and 10,763 cells, respectively. The corresponding cell numbers in choledochal cyst controls were 11,775, 5,939, 8,209, and 2,955. Cholangiocytes numbered 614 and 319 in biliary atresia and control samples, respectively, while fibroblasts numbered 963 and 98, respectively. These results suggest that the BA liver microenvironment involves immune and stromal compartments as well as the biliary epithelium. However, the relatively small cholangiocyte and especially control-fibroblast numbers should be considered when interpreting cell-type-specific differential expression results (Figure 2).

### 3.2 Biliary atresia-associated gene alterations are cell-type specific

In cholangiocytes, biliary atresia samples showed 266 upregulated genes and 457 downregulated genes (Figure 3). Representative upregulated genes included IL32, IFITM3, NEAT1, FN1, and C3, supporting inflammatory, antiviral-like, and epithelial-remodeling signals in the biliary epithelium. The heatmap of top BA-upregulated genes showed that these representative genes were most prominent in the corresponding cell-type and disease-group strata (Figure 3).

**Figure 3:**
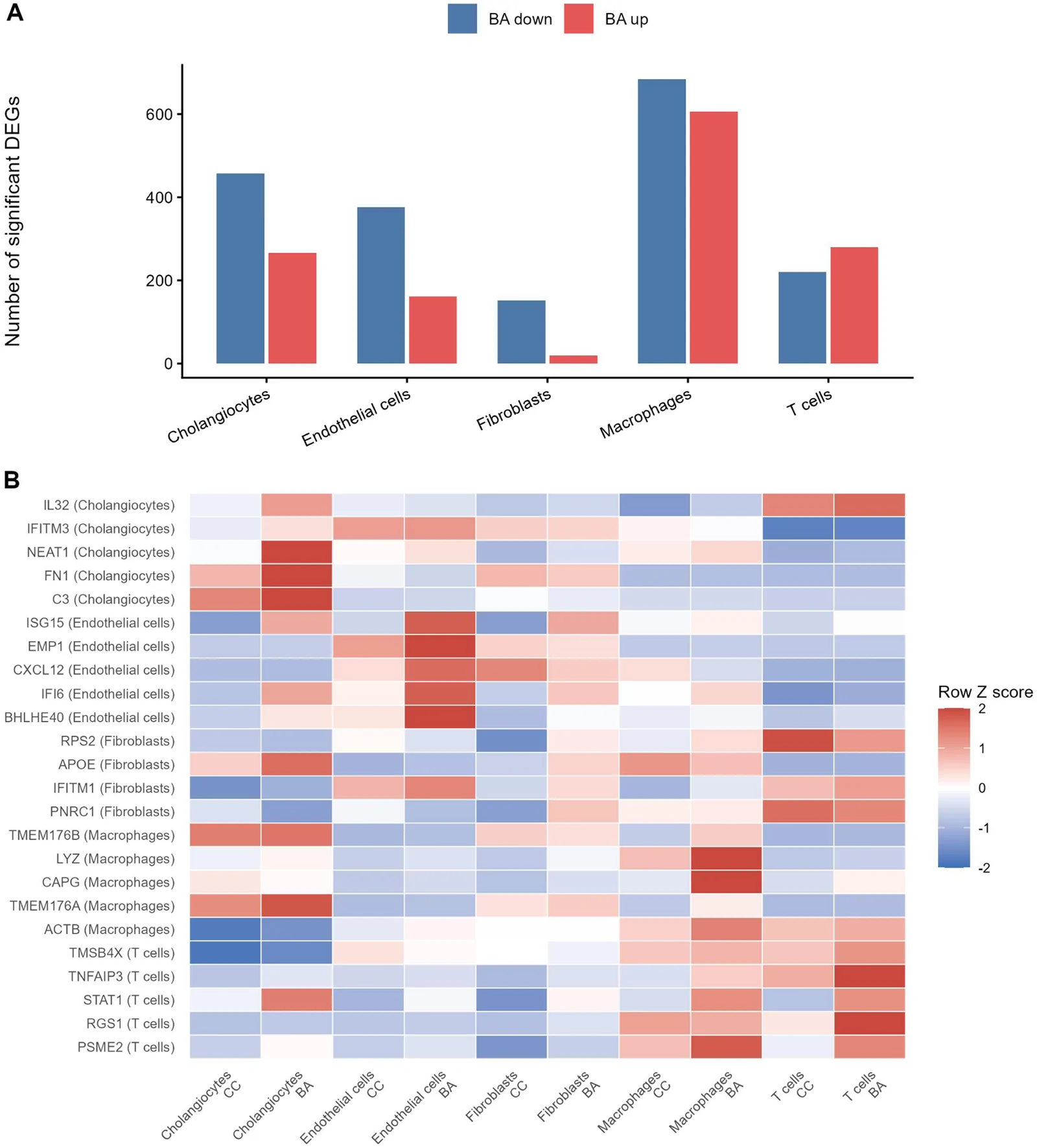
Cell-type-specific disease-associated gene alterations. **(A)** Numbers of significant BA-upregulated and BA-downregulated genes in cholangiocytes, endothelial cells, fibroblasts, macrophages, and T cells. **(B)** Heatmap showing average expression patterns of the top BA-upregulated genes selected from each major cell population.

Macrophages showed more pronounced disease-associated alterations, with 606 genes upregulated and 684 genes downregulated in biliary atresia. Upregulation of genes such as TREM2, CYP27A1, CD9, CXCL10, and APOBEC3A suggested the presence of activated inflammatory macrophage populations in biliary atresia liver tissue.

T cells contained 280 biliary atresia-upregulated genes and 220 downregulated genes. Upregulation of STAT1, DUSP4, DUSP5, FRMD4B, ENC1, and related genes indicated T-cell activation and interferon-associated response patterns in the disease microenvironment. Endothelial cells contained 161 upregulated genes and 376 downregulated genes, and increased expression of ISG15, CMPK2, OASL, and HPGD indicated that vascular-related cells also carried inflammatory-response signals.

The number of biliary atresia-upregulated genes in fibroblasts was relatively small, with only 19 genes meeting the filtering criteria, whereas 152 genes were downregulated. This limited upregulated set may reflect the strict filtering criteria, the small number of control fibroblasts, technical variability, or true stromal-state differences. Therefore, fibroblast findings were retained as candidate observations and interpreted less strongly than the cholangiocyte, macrophage, and T-cell results (Figure 3).

### 3.3 Cell-type-level enrichment highlights inflammatory and remodeling programs

Based on biliary atresia-upregulated genes from five key cell populations, a 219-gene pathological microenvironment signature was constructed, comprising 50 cholangiocyte genes, 50 endothelial cell genes, 19 fibroblast genes, 50 macrophage genes, and 50 T-cell genes. The cap of 50 genes per cell population was used to avoid dominance by cell types with larger DEG lists while retaining a representative, interpretable signature (Figure 3).

Cell-type-specific enrichment analysis of BA-upregulated DEGs showed distinct biological patterns before communication results were interpreted. Cholangiocyte-associated genes were enriched for lipid localization and transport-related terms; endothelial genes were enriched for regulation of viral processes; macrophage genes were enriched for phagocytosis, response to virus, and leukocyte migration; and T-cell genes were enriched for immune receptor signaling and actin filament organization. Fibroblast enrichment was based on only 19 BA-upregulated genes and was therefore considered exploratory. The combined 219-gene signature also supported immune and antiviral-response themes, indicating that the BA-associated microenvironment reflects inflammatory and epithelial-stromal remodeling signals rather than fibrosis alone (Figure 4).

**Figure 4:**
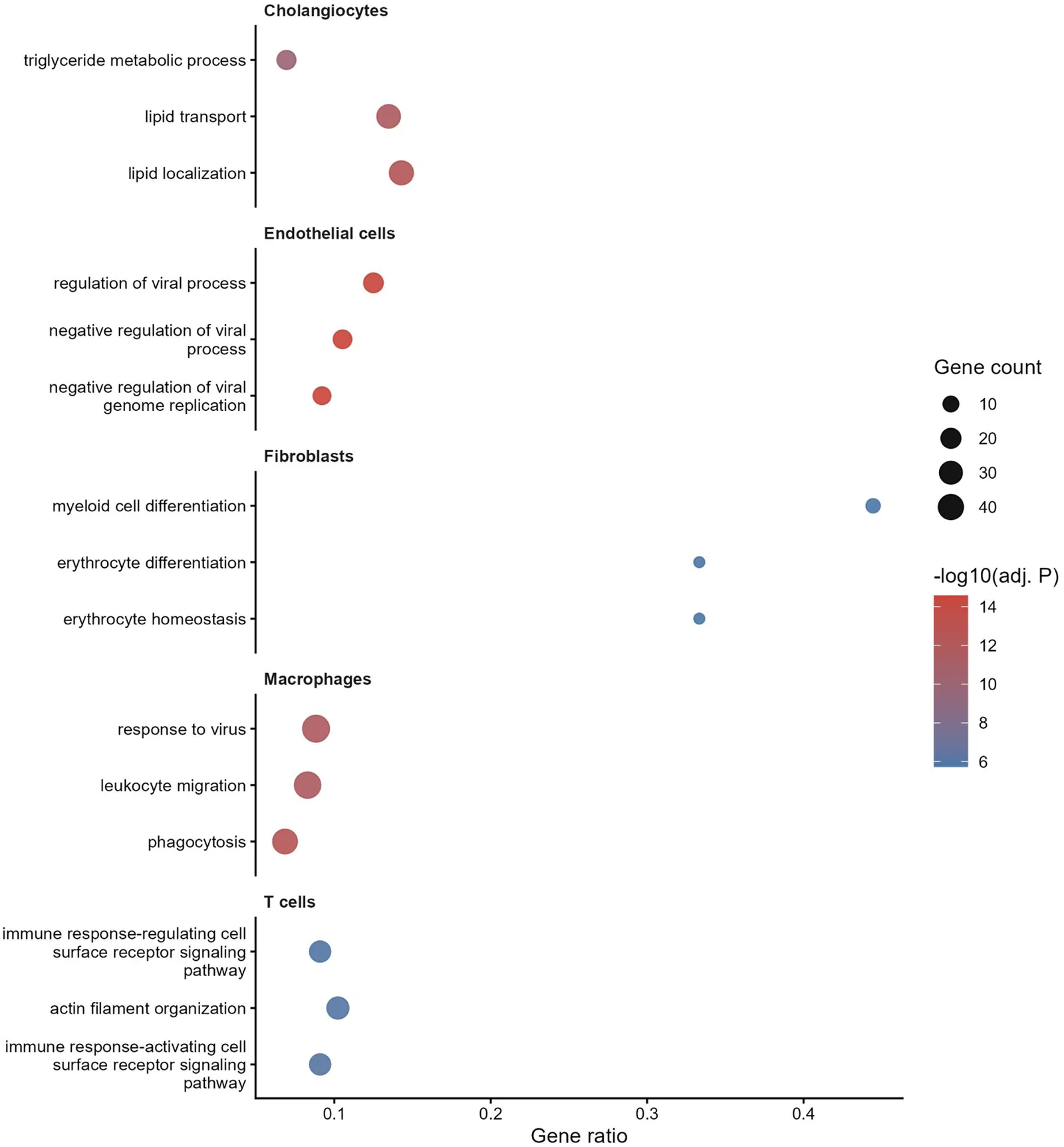
Gene ontology biological process enrichment of BA-upregulated genes in major cell populations. Enrichment patterns highlighted lipid localization and transport in cholangiocyte-associated genes, viral-response terms in endothelial genes, phagocytosis and leukocyte migration in macrophage genes, immune receptor signaling in T-cell genes, and exploratory fibroblast-associated terms based on a smaller gene set.

### 3.4 Independent bulk-tissue cohorts support the biliary atresia pathological microenvironment signature

In GSE122340, all five cell-origin signatures were higher in biliary atresia samples than in normal liver samples (Figure 5). The median values in biliary atresia vs. normal controls were 0.055 vs. −0.773 for the cholangiocyte signature (*P* = 1.01e-5), 0.010 vs. −0.745 for the endothelial cell signature (*P* = 1.13e-4), 0.027 vs. −0.783 for the fibroblast signature (*P* = 7.07e-5), 0.013 vs. -1.107 for the macrophage signature (*P* = 8.18e-6), and −0.015 vs. −0.848 for the T-cell signature (*P* = 2.35e-5). The composite microenvironment score was also increased, with a median of 0.024 vs. −0.784, *P* = 1.01e-5, and an area under the curve of 0.993 (Figure 6).

**Figure 5:**
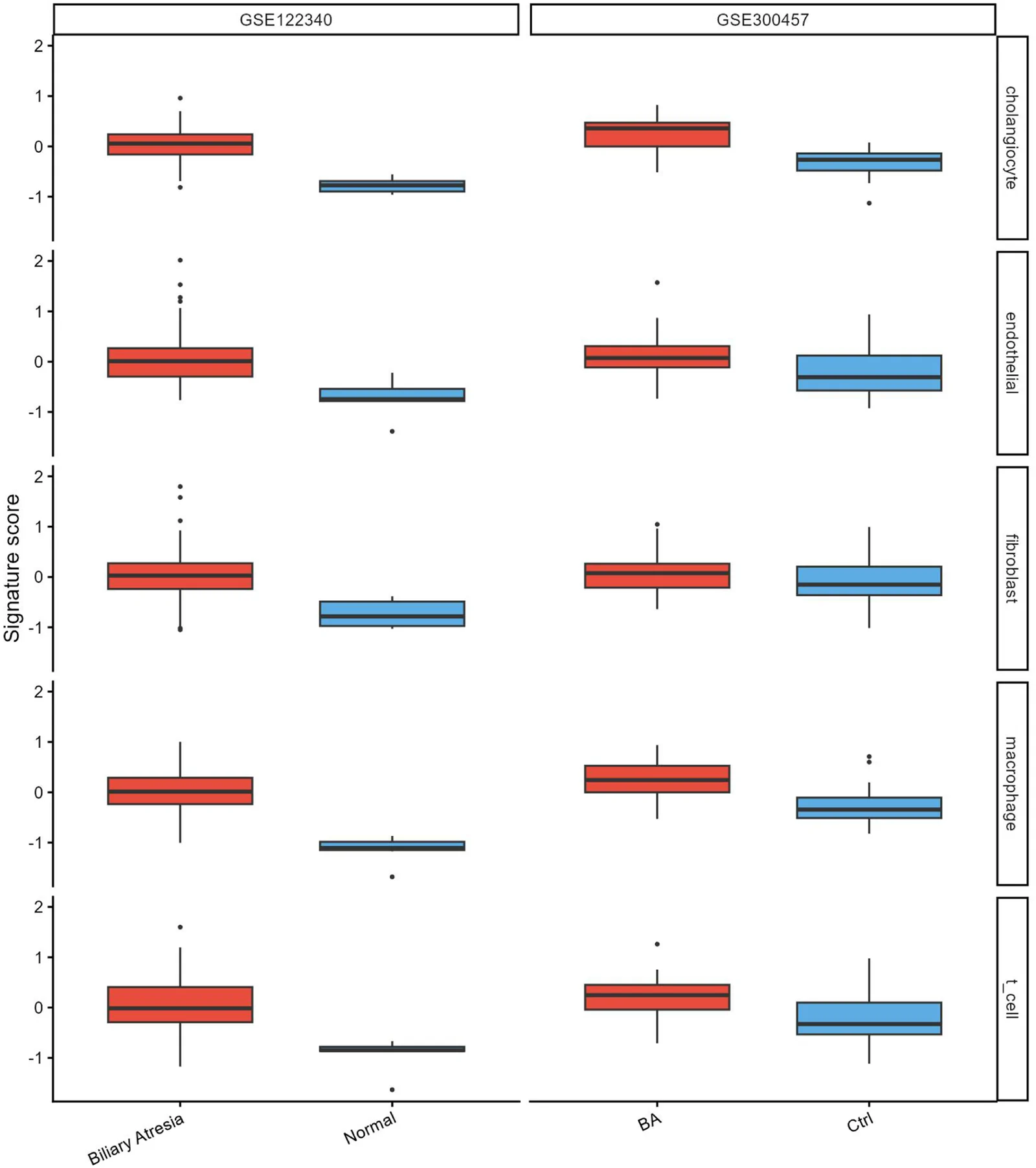
Cell-origin signature scores in independent bulk-tissue cohorts. Both GSE122340 and GSE300457 supported increased cholangiocyte-, macrophage-, T cell-, and endothelial cell-related signatures in biliary atresia.

**Figure 6:**
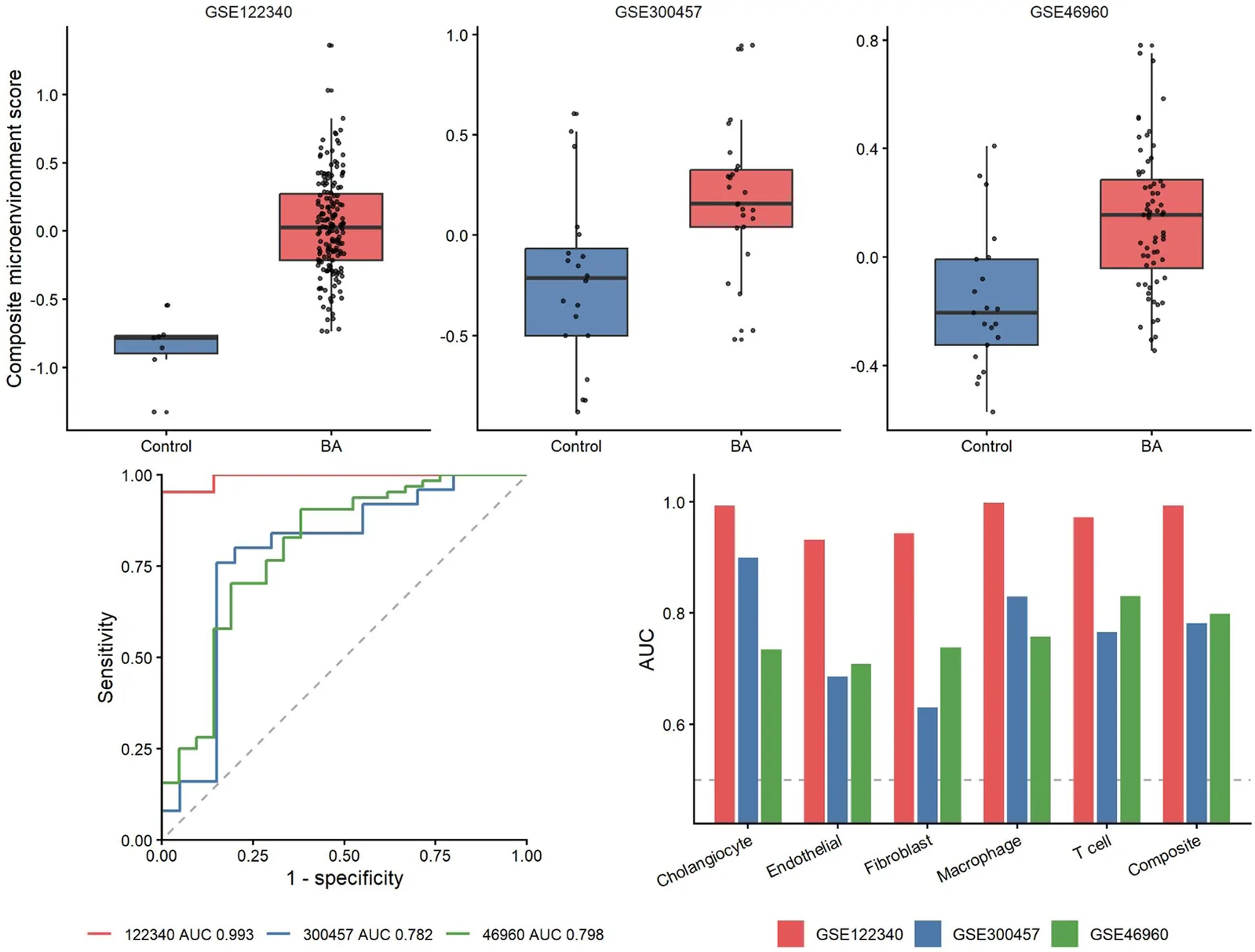
Composite microenvironment score and discriminative performance in three bulk-tissue cohorts. GSE122340, GSE300457, and GSE46960 all showed elevated composite microenvironment scores in biliary atresia samples, with areas under the curve of 0.993, 0.782, and 0.798, respectively.

In GSE300457, cholangiocyte, endothelial cell, macrophage, and T-cell signatures were also higher in biliary atresia samples than in controls. The median cholangiocyte signature was 0.358 vs. -0.266 (*P* = 7.29e-7), the endothelial cell signature was 0.072 vs. −0.309 (*P* = 0.0337), the macrophage signature was 0.243 vs. -0.343 (*P* = 8.33e-5), and the T-cell signature was 0.247 vs. −0.328 (*P* = 0.00194). The fibroblast signature showed the same direction but did not reach statistical significance, with a median of 0.075 vs. −0.152 (*P* = 0.142). The composite microenvironment score was significantly increased in biliary atresia samples, with a median of 0.157 vs. −0.214, *P* = 9.59e-4, and an area under the curve of 0.782 (Figure 6).

In GSE46960, all five cell-origin signatures were also higher in biliary atresia samples than in non-biliary atresia liver controls. The areas under the curve for cholangiocyte, endothelial cell, fibroblast, macrophage, and T-cell signatures were 0.734, 0.708, 0.738, 0.757, and 0.831, respectively, with adjusted *P* values all below 0.005. The median composite microenvironment score was 0.156 vs. −0.205 (*P* = 4.49e-5), with an area under the curve of 0.798 (Figure 6).

Together, these results indicate that cholangiocyte, macrophage, T-cell, endothelial cell, and composite microenvironment scores are consistent across liver cohorts from different sources (Figures 5, 6). In contrast, the fibroblast signature did not reach significance in GSE300457 but showed the same direction and reached significance in GSE46960, suggesting that the stromal component is less robust and may be influenced by cohort composition, control-disease type, and cell-subset complexity. A rank-based single-sample sensitivity analysis produced consistent composite-score separation across the three cohorts, supporting that the overall validation was not dependent on the mean Z-score framework. The signature scoring sensitivity summary is provided in Supplementary Table 2.

### 3.5 Cholangiocytes and fibroblasts display substantial internal heterogeneity

The cholangiocyte trajectory included 933 cells and 23,160 genes and formed six state clusters. The fibroblast trajectory included 1,061 cells and 23,160 genes and formed seven state clusters (Figure 7). When colored by disease group, both cell populations showed within-group heterogeneity, suggesting that epithelial and stromal cells in biliary atresia liver tissue are not homogeneous states.

**Figure 7:**
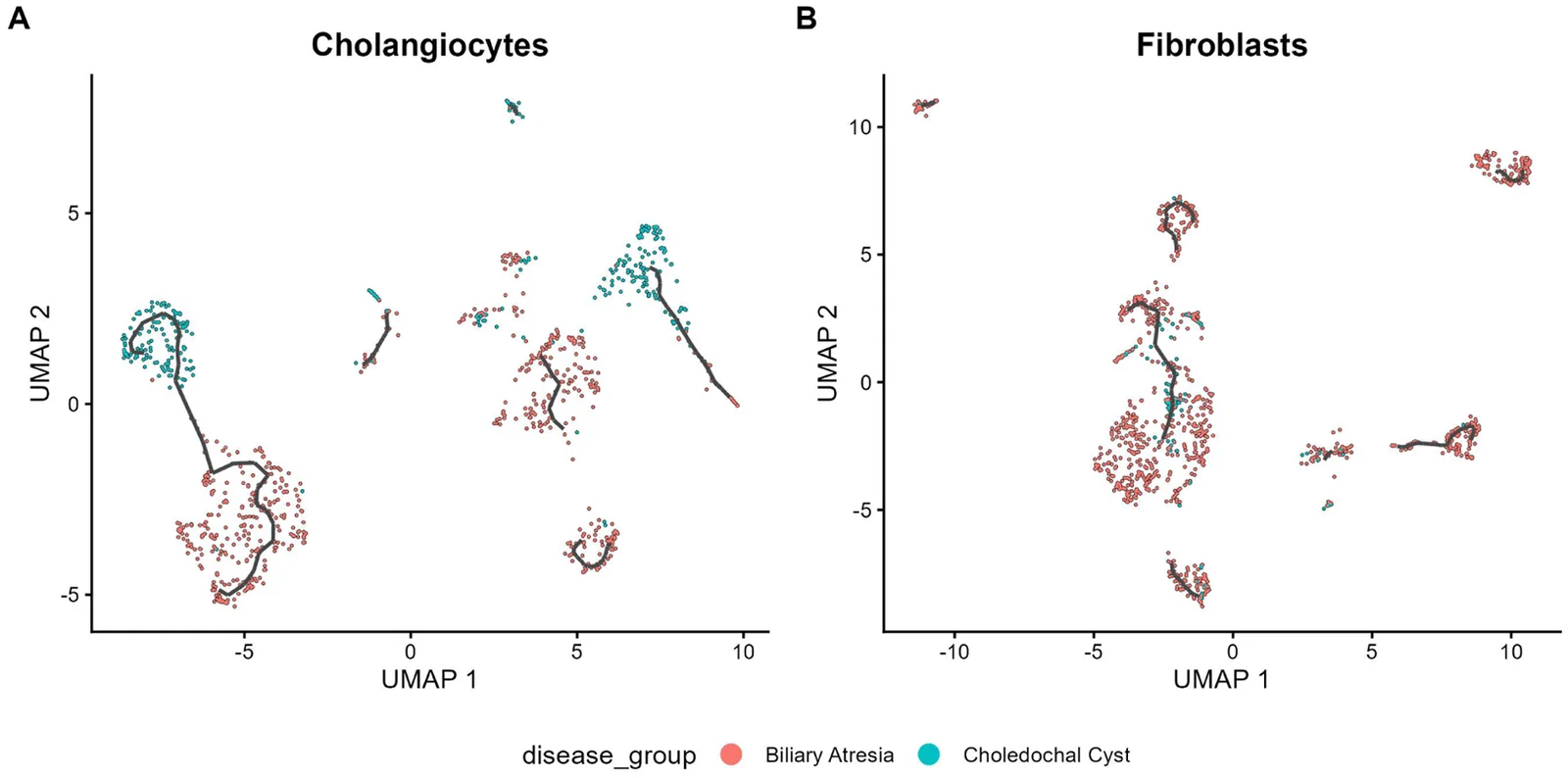
State heterogeneity of cholangiocytes and fibroblasts. **(A)** Cholangiocyte trajectory map. **(B)** Fibroblast trajectory map. The plots describe transcriptional-state heterogeneity and should not be interpreted as a definitive temporal or disease-progression direction.

Because no starting state was specified in this study, the trajectory plots should not be interpreted as a definite direction of disease progression. A more appropriate interpretation is that cholangiocytes and fibroblasts exist in multiple interconnected transcriptional states in the disease context, which may be jointly influenced by inflammation, cholestasis, and the local stromal environment. Exploratory marker-module review further supported heterogeneity within cholangiocyte, macrophage, and fibroblast compartments, but these profiles were not used to define definitive subtypes because the analysis lacked spatial confirmation and contained limited control fibroblast numbers. The exploratory substate marker profile is provided in Supplementary Table 1.

### 3.6 Cell-cell communication analysis nominates candidate inflammatory and epithelial-stromal axes

In cell-cell communication inference, 4,500 cells were included from the biliary atresia group and 3,487 cells from the choledochal cyst group (Figure 8). A total of 1,483 significant ligand-receptor interactions were identified in the biliary atresia group, compared with 1,485 in the control group. Although the overall numbers were similar, the pathway composition differed between groups.

**Figure 8:**
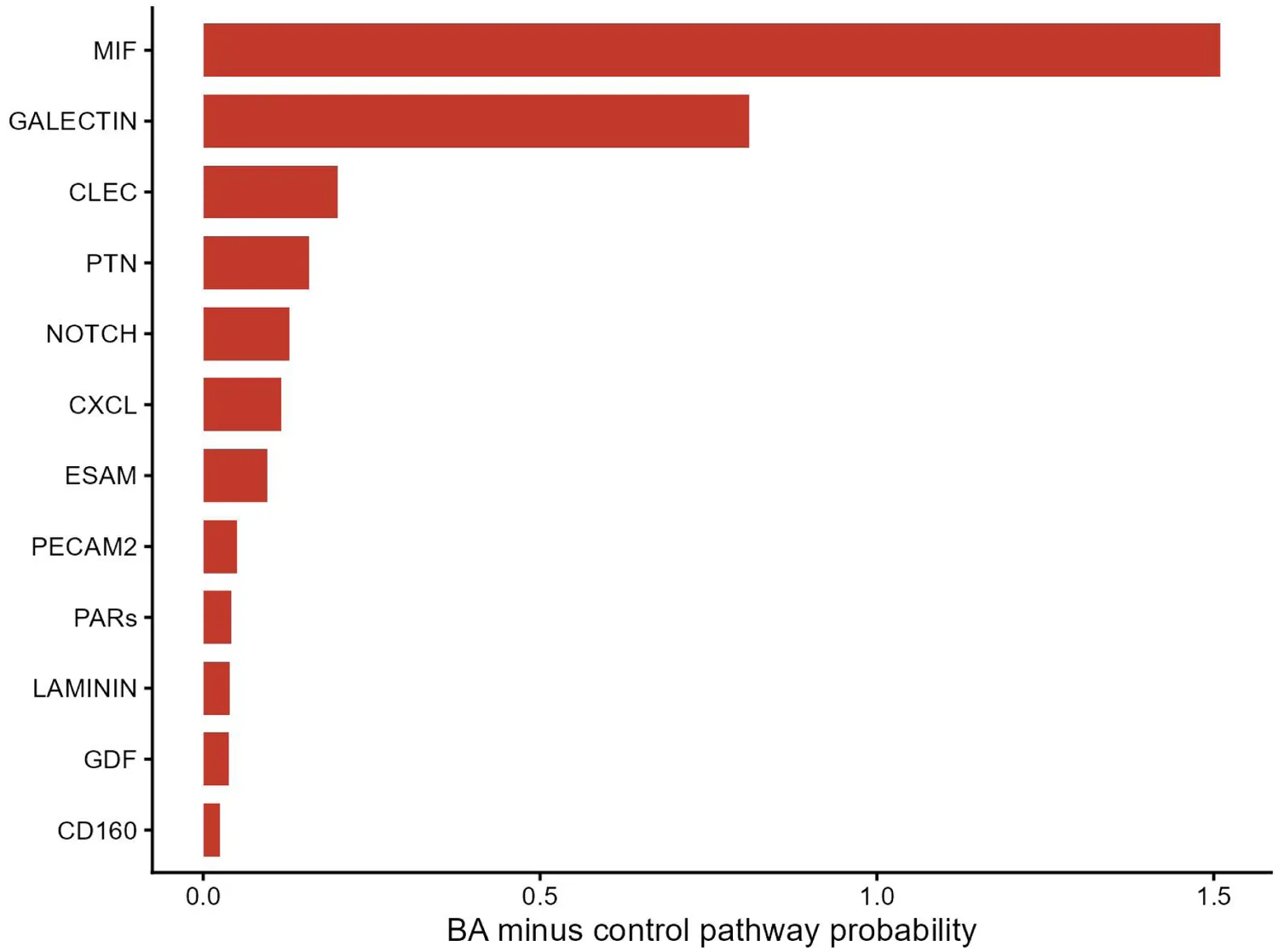
Relatively enhanced cell-cell communication pathways in biliary atresia. Signals including MIF, GALECTIN, CLEC, PTN, NOTCH, and CXCL represent candidate co-expression-based communication axes that require spatial and functional validation.

Pathways relatively enhanced in biliary atresia included MIF, GALECTIN, CLEC, PTN, NOTCH, CXCL, ESAM, PECAM2, PARs, LAMININ, GDF, and CD160. Among these, MIF, GALECTIN, CLEC, PTN, NOTCH, and CXCL showed the most prominent relative enhancement, suggesting candidate inflammatory recruitment, glycan-binding protein, epithelial-stromal, and tissue-remodeling axes for follow-up validation (Figure 8). Pathways relatively enhanced in controls included MHC-I, MHC-II, CypA, APP, VTN, CD99, MK, IGFBP, SPP1, CD45, THBS, and COLLAGEN.

Regarding communication direction, fibroblast-to-cholangiocyte interactions were prominent in both groups, with 66 significant interactions in biliary atresia and 68 in controls. In biliary atresia, additional directional interactions were inferred from smooth muscle cells to cholangiocytes, endothelial cells to endothelial cells, fibroblasts to endothelial cells, cholangiocytes to cholangiocytes, and fibroblasts to macrophages (Figure 8).

These results nominate a local microenvironment model centered on epithelial-stromal interaction and accompanied by inflammatory and chemotactic signals. MIF-, CXCL-, GALECTIN-, and NOTCH-related pathways are of particular interest because MIF signaling is linked to innate immune regulation and inflammatory amplification, GALECTIN signaling reflects glycan-binding immunoregulatory interactions, CXCL signaling is associated with leukocyte recruitment, and NOTCH signaling is relevant to epithelial-stromal communication. These axes should be regarded as prioritized hypotheses rather than confirmed mechanisms (Figure 9).

**Figure 9:**
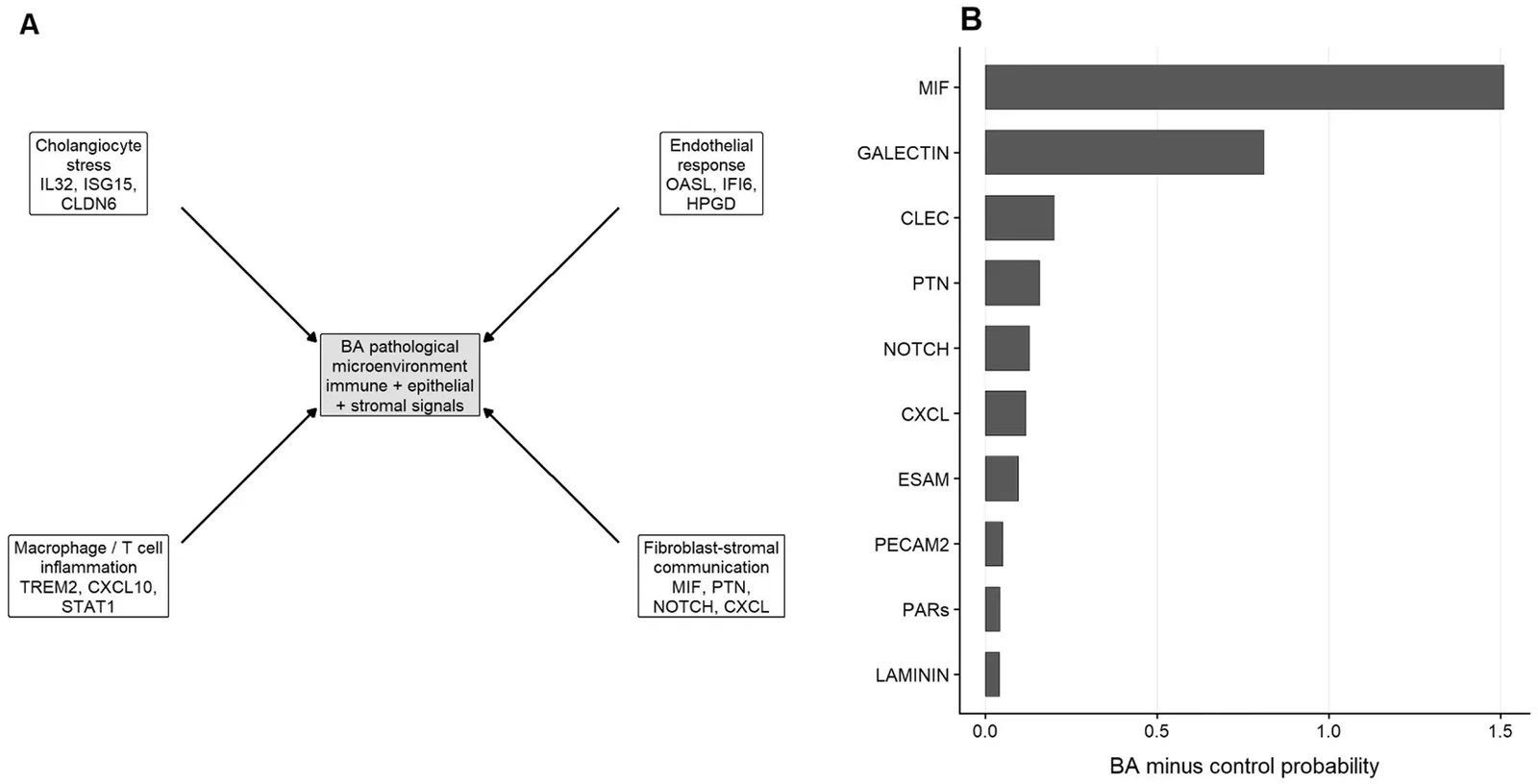
Working model of the BA-associated pathological microenvironment. Cholangiocyte stress, macrophage and T-cell inflammatory responses, endothelial involvement, and fibroblast-related communication nominate an epithelial-immune-stromal network for future validation.

## 4 Discussion

This study characterized the pathological microenvironment of biliary atresia liver from four perspectives: cell populations, gene alterations, cross-cohort consistency, and cell-cell communication. The results showed that biliary atresia-associated alterations are not limited to cholangiocytes but also involve macrophages, T cells, endothelial cells, and fibroblasts. This is consistent with the understanding of biliary atresia as a complex fibroinflammatory liver disease (21, 22). The composite microenvironment scores in three bulk-tissue cohorts supported this interpretation, indicating a degree of cross-platform reproducibility.

The cholangiocyte findings suggest that injured biliary epithelium may actively participate in the inflammatory microenvironment. Alterations in genes such as IL32, IFITM3, NEAT1, FN1, and C3, together with the stable elevation of cholangiocyte signatures in bulk-tissue cohorts, indicate that biliary epithelium in biliary atresia exists in a sustained stress- and inflammation-associated state. This interpretation is consistent with studies of cholangiopathies and the immunopathogenesis of infantile biliary atresia, which highlight the active participation of biliary epithelium in inflammatory responses (23, 24).

The macrophage and T-cell findings emphasize the central role of immune responses in biliary atresia. Increased expression of TREM2, CXCL10, STAT1, and DUSP family genes suggests that innate and adaptive immunity may jointly drive inflammatory amplification in portal areas. Previous studies of T-cell clonal expansion in target organs and liver macrophage biology support the involvement of immune cells in bile duct injury and the maintenance of fibroinflammation (25–27). The antiviral response and leukocyte differentiation terms identified by functional enrichment are also consistent with hypotheses involving virus-like triggers and immune-mediated bile duct injury.

Recent biliary atresia single-cell and spatial studies provide useful context for this candidate epithelial-stromal axis. Ye et al. described a fibrosis-related immune landscape in biliary atresia and emphasized the spatial coupling of immune and fibrotic compartments (28), while single-cell immune profiling identified coordinated immune-cell abnormalities that converge on cholangiocyte injury (29). Placed in this context, the prominent fibroblast-to-cholangiocyte communication inferred in the present study is best interpreted as a candidate portal-niche interaction rather than an isolated fibroblast signal. One plausible model is that stressed cholangiocytes release inflammatory and chemotactic cues, whereas activated fibroblasts or portal stromal cells provide extracellular-matrix remodeling, growth-factor, CXCL-related, and NOTCH-related signals that may reinforce ductular reaction, inflammatory recruitment, and portal fibrotic remodeling (36–38). Because the present analysis is based on ligand-receptor co-expression, this interpretation remains hypothesis-generating and requires spatial colocalization and functional validation.

Among candidate communication axes, MIF-, GALECTIN-, CXCL-, and NOTCH-related signals deserve priority attention for validation. MIF is involved in innate immune regulation and inflammatory amplification, whereas GALECTIN-related glycan-binding protein signaling is closely linked to immune tolerance and inflammatory regulation (30, 31). However, ligand-receptor analysis can only infer potential cellular interactions based on co-expression patterns, and its level of evidence is lower than that of spatial colocalization and functional perturbation experiments. Thus, these pathways should not be interpreted as proven causal drivers. This limitation also applies to related frameworks such as CellPhoneDB and NicheNet (32, 33).

This study has several limitations. First, it was based on public cohorts and lacked histological validation using samples from our own center. Second, the main single-cell control group consisted of choledochal cyst rather than normal pediatric liver tissue. Therefore, some signals described here may reflect cholestasis, biliary injury, or shared fibroinflammatory responses across biliary diseases rather than BA-specific mechanisms. Third, although exploratory marker review suggested heterogeneity within cholangiocyte, macrophage, and fibroblast compartments, the main analysis was intentionally kept at the broad cell-type level because control cholangiocyte and fibroblast numbers were limited and spatial validation was unavailable. Fourth, ligand-receptor relationships can only suggest potential cellular interactions and cannot directly prove signal activation, spatial proximity, or functional relevance. Animal models and recent immunological studies suggest that interferon-associated bile duct injury and CD8+ tissue-resident memory T cells may be involved in biliary atresia progression (34, 35).

Overall, biliary atresia liver shows a BA-associated pathological microenvironment involving cholangiocyte stress, immune-cell activation, endothelial participation, and stromal communication. The cross-cohort composite score strengthens the reproducibility of this observation, although it does not establish disease specificity by itself. The most actionable follow-up hypotheses are to test whether MIF-CD74/CXCR4, CXCL-related chemotactic signaling, GALECTIN signaling, and NOTCH-related epithelial-stromal interactions are spatially colocalized in portal areas and whether their signal intensity correlates with fibrosis severity or postoperative clinical outcome.

## 5 Conclusion

This study identified an epithelial-immune-stromal microenvironment signature associated with biliary atresia relative to available public controls. Cholangiocyte, macrophage, T-cell, endothelial cell, and composite microenvironment scores showed consistency across independent liver cohorts, whereas fibroblast findings were less robust. Cell-cell communication results nominate fibroblast-to-cholangiocyte interactions and MIF, GALECTIN, CXCL, and NOTCH signaling as candidate axes for subsequent spatial, histological, and functional validation.

## Data Availability

The datasets analyzed in this study are publicly available in the Gene Expression Omnibus under accession numbers GSE176189, GSE122340, GSE300457, and GSE46960. Other data generated or reviewed in this study are included in the article and Supplementary Material; further inquiries can be directed to the corresponding author.

## Supplementary material

Supplementary Table 1 Exploratory substate marker profile by cluster.

Supplementary Table 2 Signature scoring sensitivity summary.
